# Hepcidin, an emerging and important player in brain iron homeostasis

**DOI:** 10.1186/s12967-018-1399-5

**Published:** 2018-02-07

**Authors:** Driton Vela

**Affiliations:** Department of Physiology, Faculty of Medicine, University of Prishtina, Martyr’s Boulevard n.n., 10000 Prishtina, Kosova

**Keywords:** Hepcidin, Astrocytes, Inflammation, Brain hemorrhage, Alzheimer’s disease

## Abstract

Hepcidin is emerging as a new important factor in brain iron homeostasis. Studies suggest that there are two sources of hepcidin in the brain; one is local and the other comes from the circulation. Little is known about the molecular mediators of local hepcidin expression, but inflammation and iron-load have been shown to induce hepcidin expression in the brain. The most important source of hepcidin in the brain are glial cells. Role of hepcidin in brain functions has been observed during neuronal iron-load and brain hemorrhage, where secretion of abundant hepcidin is related with the severity of brain damage. This damage can be reversed by blocking systemic and local hepcidin secretion. Studies have yet to unveil its role in other brain conditions, but the rationale exists, since these conditions are characterized by overexpression of the factors that stimulate brain hepcidin expression, such as inflammation, hypoxia and iron-overload.

## Background

Hepcidin has been subject of increased interest from researchers ever since its discovery in 1998, and especially since 2001, when Pigeon et al. discovered its crucial role in iron homeostasis [1]. The most important data about its actions and regulation come from studies that have examined the liver isoform of this peptide. Liver hepcidin is the most abundant source of systemic hepcidin, but data suggest that not only hepcidin is produced locally in different organs, but local isoforms can serve important functions for organ homeostasis as well [2–4].

## Systemic iron metabolism

Humans secure iron from different food sources. Dietary iron is absorbed through the intestines, that is, enterocytes, after being reduced by apical ferrireductases [5]. Divalent iron then enters in enterocytes through divalent metal transporter 1 (DMT1). Inside enterocytes iron is transported via poly-(rC)-binding proteins (PCBPs) to iron depots (ferritin) and molecules that use iron as a co-factor [6]. It is still not entirely known if PCBPs deliver iron to ferroportin (FPN), but we know that FPN acts as the major iron-exporter out of cells [5, 6]. During its efflux out of enterocytes iron is oxidized and immediately bound to transferrin (Tf) [5]. Tf-iron complex circulates in the blood and finally binds with transferrin receptor 1 (TFR1) receptor in target cells. Cellular iron availability is controlled by iron regulatory element/iron regulatory protein (IRE/IRP) system, which is reactive to cellular iron concentrations [5, 6]. IRE/IRP is able to control cellular iron homeostasis by controlling the expression of cellular iron importers and exporters [5, 6]. It is evident that in every stage of iron transport, iron tends to be bound with different proteins, which serves as a protective mechanism that does not allow iron access to pathogenic microbes [5]. In addition, iron is a highly reactive element which might cause oxidative stress in our cells, therefore the chemical modification of iron by reductases and its transport realized through binding with different proteins, like ferritin (intracellularly) and Tf (extracellularly) protects tissues from oxidative damage [5, 6].

### Liver hepcidin acts as a major regulator of systemic iron metabolism

**Fig. 1:**
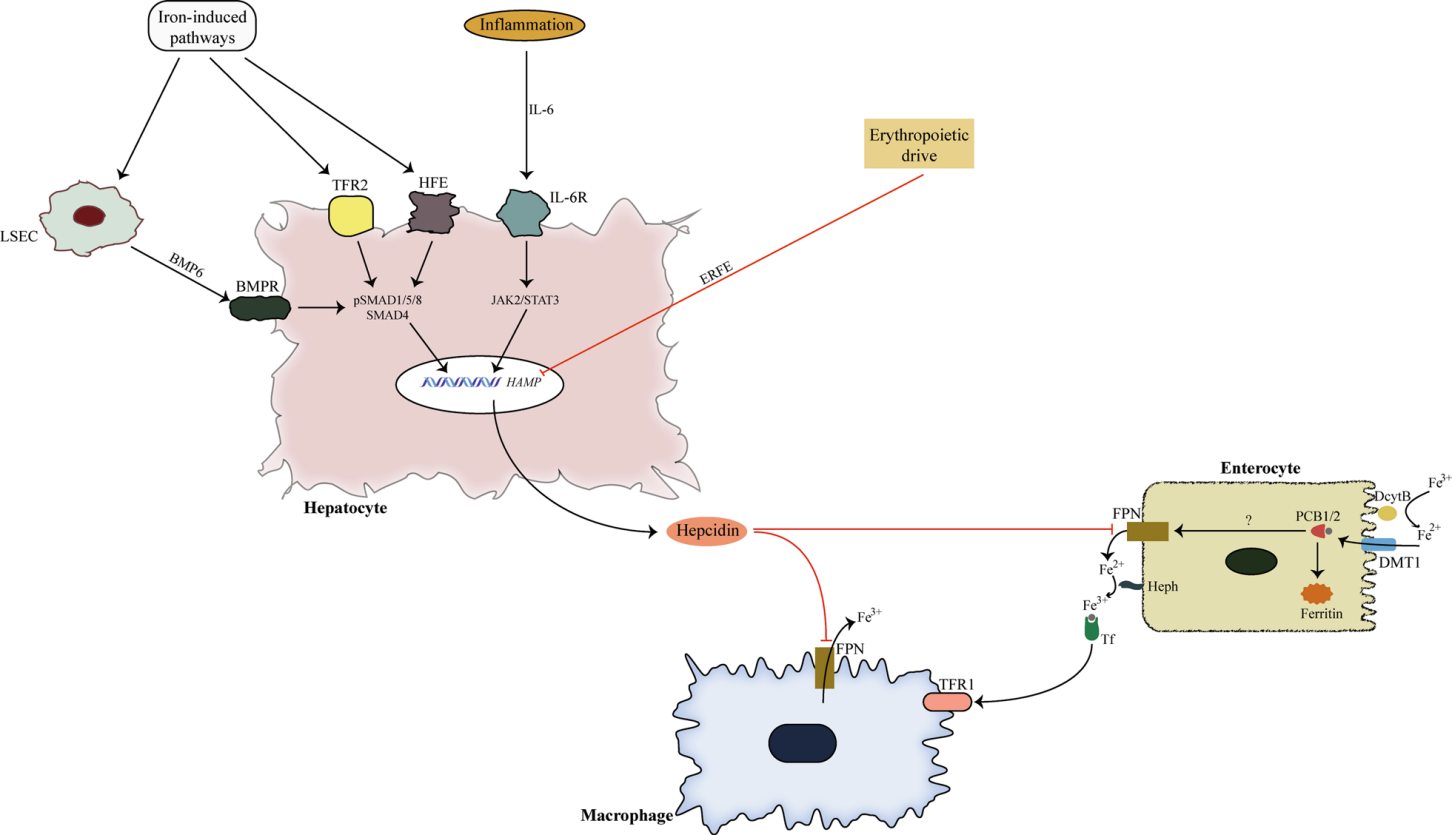
Systemic iron homeostasis. Trivalent iron is reduced by ferrireductases (DcytB) before its absorption through DMT1 in enterocytes. Once inside enterocytes iron binds with chaperones like PCBs. PCBs act like intracellular iron transporters that distribute this metal to ferritin depots and probably to FPN. FPN is the main exporter of iron out of cells. This action of FPN is helped by ferrioxidases (like Heph). After its export out of cells, iron is immediately bound to Tf. This complex circulates in plasma and finally binds with its target, which is TFR1. Systemic iron availability is controlled by hepcidin. Hepcidin is produced in hepatocytes in response to different stimuli. Iron-mediated pathways are the main factors that induce hepcidin expression. The most important pathways activate LSECs, which in turn produce BMP6. BMP6 acts in a paracrine manner through BMPR in hepatocytes. BMPR activates SMAD pathway, which induces hepcidin expression. Iron pathways induce hepcidin expression through membrane proteins, like TFR2 and HFE, as well. Inflammatory signals are also important upregulators of hepcidin by acting through JAK/STAT pathway. Negative control is realized through ERFE, which is produced from erythrocyte precursors. *BMP6* bone morphogenetic protein 6, *BMPR* BMP receptor, *DcytB* duodenal cytochrome B, *DMT1* divalent metal transporter 1, *ERFE* erythroferrone, *FPN* ferroportin, *HAMP* hepcidin antimicrobial peptide, *Heph* hephaestin, *HFE* hemochromatosis protein, *JAK2/STAT3* janus kinase 2/signal transducer and activator of transcription 3, *LSEC* liver sinusoidal endothelial cells, *PCB* poly-(rC)-binding protein, *SMAD* S-mothers against decapentaplegic, *TFR* transferrin receptor

Hepcidin is a small peptide with antimicrobial properties. It affects systemic iron availability by controlling FPN expression post-translationally [5]. It is mostly produced by liver sinusoidal endothelial cells (LSECs) in response to iron-load [7]. Iron-load induces production of bone morphogenetic protein 6 (BMP6) from LSECs, which then acts in neighbouring hepatocytes through BMP receptor (BMPR) [8] (Fig. 1). BMPR creates a supercomplex with hemojuvelin (HJV), matriptase 2 (MT2) and neogenin [9]. This scaffold of molecules controls the activity of BMPR. Once activated, BMPR induces phosphorylation of s-mothers against decapentaplegic (SMAD) molecules, which then cause an increase in hepcidin expression through activation of hepcidin antimicrobial peptide (*HAMP*) gene [5]. Iron-mediated pathways induce hepcidin expression through other membrane proteins, such as TFR2 and HFE, with HFE being less important in this context [10]. Other positive stimuli that control hepcidin expression are inflammatory stimuli, which act through janus kinase 2/signal transducer and activator of transcription 3 (JAK2/STAT3) pathway [5]. Overactivity of inflammatory cytokines is responsible for anemia of inflammation in different chronic diseases and cancer [5, 11]. Negative control of hepcidin expression is exerted by erythroferrone (ERFE) which is produced by erythrocyte precursors in order to secure more iron for erythropoiesis [12]. Other factors that have been shown to affect hepcidin expression include vitamin D, hypoxia, heparin, estrogens [13–16].

Hepcidin produced by the liver acts on its target cells, like enterocytes, macrophages, hepatocytes, and as recent data suggest, in brain cells as well [17, 18].

## Brain iron metabolism

Iron turnover in the brain is slow compared to other organs [19]. The process of iron uptake starts in the level of blood–brain barrier (BBB), where first cells to come into contact with Tf-iron complex are brain microvascular endothelial cells (BMVECs) [20]. Tight junctions between these cells preclude an iron-entry through paracellular pathways, leaving the option of transcellular pathway as the most viable one. In this way BMVECs exert a strict control on brain iron-uptake by regulating the level of receptors through which iron enters and exits BBB [20]. Tf-iron enters BMVEC through its receptor, that is, TFR [21]. Blocking TFR with specific antibodies reduces significantly the ability of BMVEC to transport iron [22]. Nevertheless, Tf-iron complex with TFR creates an endosome which will then release the reduced iron into cytoplasm via DMT1 [20, 21]. DMT1 is believed to be also responsible for non-transferrin bound iron (NTBI) entry into BMVEC by serving as a direct iron importer in the luminal surface of BMVEC [20]. Both sources of iron exit BMVEC through FPN [20]. Activity of BMVEC FPN is dependent on the presence of two ferrioxidases, hephaestin (heph) and ceruloplasmin (CP) [20]. Although iron distribution from BMVEC to brain tissue cells is not yet clear, it seems to be rather effective since systemic iron deficiency does not cause significant reduction in brain iron depots [23]. In-vivo experiments show that astrocytes are important mediators of iron transport from BMVEC to brain tissue [21]. They secrete ferroxidases that stabilize BMVEC FPN, but also are an important source of local hepcidin through which they control iron entry into brain tissue [21, 24]. Astrocytes are also important local regulators of neuron homeostasis by sequestering excess iron during iron-overload [25–27]. After crossing the BBB barrier iron is used for its metabolic needs by neurons. It enters neuronal cells through TFR and DMT1 [28]. These two proteins are believed to be more prominently expressed in neurons compared to glial cells [29]. Recent data suggest that transport of NTBI in brain cells is dependent on newly described proteins, that is, Zip8 and Steap2 [30], though the exact role of these proteins in brain iron metabolism remains to be elucidated.

## Hepcidin production and action in the brain

First studies that have examined hepcidin expression in the brain yielded somewhat different results. According to Krause et al. hepcidin expression in the brain is low but still third next to liver and heart [31]. Other studies found that this expression was even lower than the previous study suggested [1, 32]. In another study, which examined mRNA and protein levels of hepcidin, results showed a marked discrepancy between these two markers of hepcidin presence, with protein levels being more pronounced than mRNA levels of hepcidin [33]. These results were similar with observations from Zechel et al. study [32], where data from in situ hybridization (which measured mRNA expression) did not match results from immunohistochemistry (which measured protein distribution) (Table 1). These data imply that at least some of hepcidin in the brain in fact comes from liver [33]. mRNA of hepcidin is not uniformly expressed in different regions of rat brain; higher mRNA is observed in cortex, striatum, cerebellum [32, 34], while others failed to detect mRNA in cortex [33], though protein levels of hepcidin were found in choroid plexus, corpus callosum, olfactory bulb, cortex, subventricular zone [33, 35]. The differences in mRNA expression seem to have been related to the sensitivity of the methods used to detect hepcidin expression (RT-PCR vs in situ hybridization). Hepcidin protein has also been observed in mature astrocytes, especially in those localized in the walls of lateral ventricle [35]. Chowdhury et al. study has revealed that hepcidin protein is present in glial cells, but not in mature neurons [35]. All these observations show that hepcidin expression in the brain is low in basal conditions. The presence of hepcidin in vascular structures and cellular components of BBB shows that the hepcidin role in basal conditions might include the control of iron entry from plasma into the brain. But, it seems that systemic hepcidin is redundant in controlling brain iron homeostasis in basal conditions. In liver (systemic) hepcidin knockout mice brain iron is not significantly altered [18]. In hemochromatosis (HH), which is associated with hepcidin disturbance, brain iron-load is a seldom occurrence [36], mostly present when accompanied with abnormally high levels of ferritin and transferrin saturation, or liver cirrhosis, in which condition iron can pass more freely in brain parenchyma due to BBB disruption [37–39]. In conclusion, systemic hepcidin loss not accompanied by additional deleterious factors does not seem to cause significant brain iron-load. This is further strengthened by observations that brain iron-overload in HH is more frequently present in progressive and terminal stages of the disease [38]. Since iron is indispensable for brain homeostasis, neurons have evolved important strategies to counteract local iron disturbance. During iron-overload neurons increase ferritin to sequester iron in nontoxic form, downregulate iron importers and increase iron export [40]. Furthermore, astrocytes increase their iron-import during iron-overload to protect neurons from damage [27]. Intrinsic iron regulation in the brain does seem to compensate for the loss of systemic hepcidin. Similar protective mechanisms have been observed in retina as well.

**Table 1:** Expression, regulation and action of brain hepcidin

Methods	Study conditions	Main results	References
Expression measured with RT-PCR from human tissues	Physiological conditions	mRNA of hepcidin present in brain tissue	Krause et al. [31]
Expression measured with RT-PCR from human tissues	Physiological conditions	mRNA of hepcidin present in spinal cord	Pigeon et al. [1]
Expression measured with RT-PCR, in situ hybridization, immunohistochemistry in murine brain	Physiological conditions	Hepcidin expression detected with RT-PCR and in situ hybridization Immunohistochemistry showed a wide distribution of the protein across the brain tissue compared to narrow distribution of mRNA of hepcidin measured by in situ hybridization	Zechel et al. [32]
Expression measured with RT-PCR and in situ hybridization, while protein levels were measured with western blotting and immunohistochemistry (rat brain samples)	Physiological conditions	Hepcidin expression was low with highest signal detected in choroidal plexus and to a lesser extent in blood vessels Protein levels were abundant, especially in blood vessels, choroidal plexus and cortical astrocytes in close proximity to blood vessels	Raha-Chowdhury et al. [33]
Protein levels measured with western blotting and immunohistochemistry (mouse brain samples)	Physiological conditions	Abundant protein levels of hepcidin across the brain tissue	Raha et al. [35]
Expression measured with RT-PCR, while protein levels were measured with western blotting (mouse brain and cell cultures)	Physiological and stimulated conditions	Hepcidin expression was abundant but still in low levels Hepcidin induced FPN downregulation in mouse brain Hepcidin decreases iron-release in cultured neurons due to FPN downregulation	Wang et al. [34]
Expression measured with RT-PCR (human brain samples)	Physiological conditions	HJV mRNA was not observed Hepcidin, neogenin, TFR2, HFE mRNA was present	Hänninen et al. [69]
Expression measured with RT-PCR, while protein levels were measured with western blotting (mouse brain)	Different stimulated conditions	TFR2 loss does not affect brain hepcidin protein levels	Pellegrino et al. [73]
Expression measured with RT-PCR, while protein levels were measured with western blotting and immunohistochemistry (rat cultured cells)	Inflammation	Microglia react to LPS by producing IL-6 Astrocytes produce hepcidin in response to IL-6 secreted by microglia Hepcidin increases iron-load in neurons Inflammation has hepcidin-independent actions in reducing iron-export and enhancing iron-import in neurons	You et al. [54]
Expression measured with RT-PCR, while protein levels were measured by western blotting and immunohistochemistry (rat cultured cells)	Inflammation and iron-stimulation	TNF-α, IL-6 and LPS increase cellular iron content in neurons TNF-α, IL-6 and LPS do not affect cellular iron content in astrocytes TNF-α, IL-6 and LPS increase cellular iron content in microglia TNF-α, IL-6 and LPS cause DMT1↑ and FPN↓ (protein levels) in neurons TNF-α, IL-6 and LPS cause DMT1↑ (protein levels) in astrocytes TNF-α, IL-6 and LPS cause DMT1↑ (protein levels) in microglia TNF-α, IL-6 and LPS cause DMT1↑ (mRNA) in neurons TNF-α, IL-6 and LPS cause DMT1↑ (mRNA), while LPS causes FPN↓ in astrocytes TNF-α, IL-6 and LPS cause DMT1↑ (mRNA), while TNF-α and LPS cause FPN↓ (mRNA) in microglia TNF-α, IL-6, LPS and FeNTA cause hepcidin↑(mRNA) in astrocytes and microglia but not in neurons	Urrutia et al. [56]
Protein levels measured with western blotting and ELISA in rat cultured cells	Inflammation	LPS increases hepcidin expression in rat brain LPS increases IL-6 production from microglia IL-6 from microglia induces hepcidin production in neurons (via STAT3 pathway) No observed hepcidin↑ in neurons without co-cultured microglia due to LPS stimulation	Qian et al. [55]
Expression measured with RT-PCR, while protein levels were measured with western blotting and immunohistochemistry (rat brain and cultured cells)	Iron-overload	Hepcidin↑ in rat brain and cultured cells Hepcidin mRNA↑ in cultured neurons in response to iron-overload	Sun et al. [64]
Expression measured with RT-PCR, while protein levels were measured with western blotting and ELISA in rat brain and cultured cells	Iron-overload	Hepcidin reduces iron-load in rat brains Hepcidin induces TFR1↓, DMT1↓ and FPN↓ in BMEC Hepcidin induces TFR1↓, DMT1↓ and FPN↓ in neurons	Du et al. [59]
Expression measured with RT-PCR, while protein levels were measured with western blotting and ELISA in rat brain and cultured cells	Physiological conditions	Hepcidin induces TFR1↓, DMT1↓ and FPN↓ in astrocytes TFR1↓ is realized through AMPK pathway	Du et al. [58]
Expression measured by RT-PCR, while levels of chemicals were measured with ELISA	Inflammation induced by Aβ aggregates	Hepcidin pre-treatment reduces expression and secretion of IL-6 and TNF-α in astrocytes and microglia	Urrutia et al. [65]

*Aβ* amyloid beta, *AMPK* AMP-activated protein kinase, *BMEC* brain microvascular endothelial cells, *DMT1* divalent metal transporter 1, *ELISA* enzyme-linked immunosorbent assay, *FeNTA* ferric nitrilotriacetate, *FPN* ferroportin, *HFE* hemochromatosis protein, *HJV* hemojuvelin, *IL-6* interleukin 6, *LPS* lipopolysaccharide, *RT-PCR* reverse transcription polymerase chain reaction, *STAT3* signal transducer and activator of transcription 3, *TFR* transferrin receptor, *TNF-α* tumor necrosis factor-alpha

Though hepcidin is present in all the cells that comprise the BBB, less is known about the hepcidin transport through BBB. Defensins and amphipathic peptide cations similar to hepcidin are known to cross BBB [41, 42]. Hepcidin transport through BBB has not been observed directly, but hepcidin protein has been detected in the brains of mice with knockdown of systemic hepcidin after being transfused with hepcidin [18].

More consistently hepcidin expression has been detected in special function neurons such as retinal photoreceptors, but also in retinal pigment epithelium (RPE) and adjacent Muller cells [43]. Similar to LSECs in the liver, RPE produces BMP6 in response to iron-load which then induces hepcidin expression in neurosensory retina [44, 45]. Interestingly, neurosensory retinal cells express more abundant BMP receptor expression compared to RPE, suggesting that BMP6 from RPE acts on BMPR of neurosensory retinal cells. HJV seems to be important in inducing hepcidin expression in RPE, since loss of HJV in RPE cells blunts the hepcidin expression due to iron-load [46]. Hepcidin produced by retinal cells during iron-overload acts in a paracrine manner by degrading FPN in neighbouring vascular endothelial cells, thus preventing retinal iron import [45]. On the other hand, eye injury does induce hepcidin expression in retinal cells through interleukin-6 (IL-6) mediation [45]. Furthermore, in vivo and in vitro studies have shown that inflammatory stimuli induce hepcidin expression in RPE and neural retina [43]. Retinal hepcidin overexpression due to inflammatory stimuli is accompanied with cellular iron-overload and increased oxidative stress [43]. Inflammatory signaling in retina induces hepcidin independent of HFE and HJV. This action is mediated through activation of Toll-like receptor 4 (TLR4) in RPE [43]. Systemic hepcidin does not seem to have an important role in retina in basal conditions, which is suggested by the presence of significant iron-overload in retina only in aged rats with loss of hepcidin and HJV [45, 46]. It has to be mentioned that in juvenile HH caused by a dramatic loss of hepcidin expression, organ iron-overload (heart, liver, gonads) is observed early in life [47]. Incidence of retinal damage in HH is low [48], while mice models with FPN knockout show early and more prominent retinal damage compared to systemic hepcidin knockout [49]. This suggests that local FPN in retina is more important for iron homeostasis than systemic hepcidin levels.

### Hepcidin regulation in the brain

**Fig. 2:**
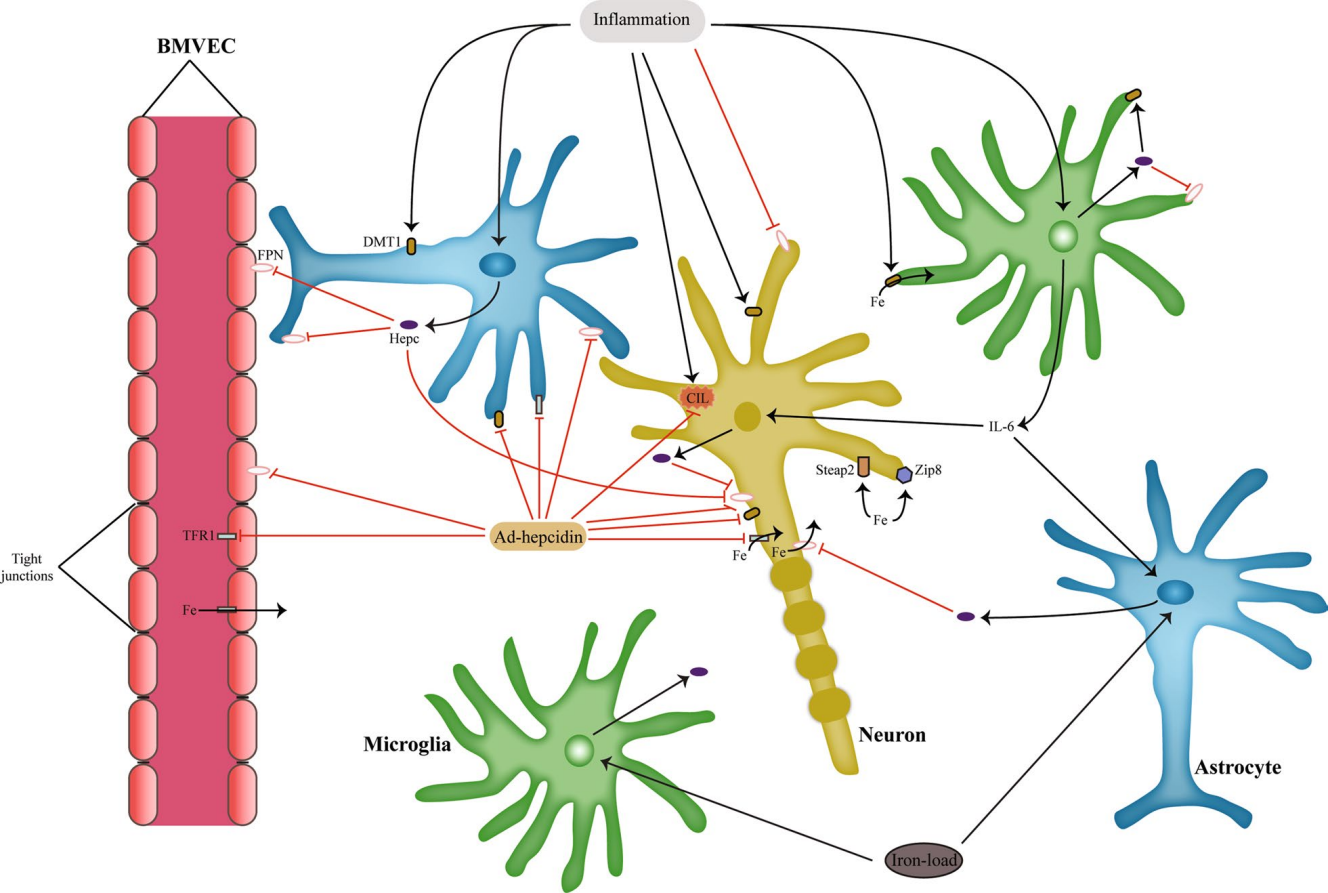
Hepcidin regulation and action in brain cells. Hepcidin expression in the brain is often induced by inflammatory stimuli. Inflammatory cytokines increase iron import through DMT1, and decrease iron export due to FPN downregulation. This increases cellular iron-load, especially in neurons. During iron-load conditions, astrocytes and microglia have been shown to increase hepcidin production. This might be the case for neurons as well, but the data are still inconclusive. Use of ad-hepcidin protects neurons during iron-overload conditions, by controlling the activity of iron import and export proteins, like TFR1, DMT1, FPN. Also, ad-hepcidin reduces iron flux from BMVEC, which reduces brain iron-load. Recent data suggest an important role for Zip8 and Steap2 for NTBI entry into brain cells. *BMVEC* brain microvascular endothelial cell, *CIL* cellular iron-load, *DMT1* divalent metal transporter 1, *FPN* ferroportin, *Hepc* hepcidin, *IL-6* interleukin 6, *NTBI* non-transferrin bound iron, *TFR1* transferrin receptor 1

Inflammation is an important inducer of local brain hepcidin expression. It can induce a significant increase in brain hepcidin expression (even up to 40-fold increase during *E. coli* infection) [50]. In epithelial cells of the choroidal plexus injection of bacterial lipopolysaccharide (LPS) induces upregulation of IL-6 and *HAMP* [51]. IL-6 upregulation is accompanied with expected STAT3 upregulation, which causes an increase in hepcidin expression [51]. Interestingly, LPS causes increased SMAD4 expression as well, which may be related with increased TFR2 expression [51]. These changes accompanied with increased ferritin expression suggest that inflammatory stimuli prevent iron export into cerebrospinal fluid (CSF) and cause iron accumulation in epithelial cells of choroidal plexus. LPS and IL-6 have consistently been related with increase in hepcidin expression in brain parenchyma as well [50, 52, 53]. Experiments with cell cultures have shown that LPS induces IL-6 expression in microglia, which then induces hepcidin production in astrocytes and probably neurons [54, 55]. LPS can induce hepcidin production in microglia as well [56, 57], while IL-6 was shown to increase hepcidin expression in neurons through phosphorylation of STAT3 [55]. Studies with cell cultures have revealed that, in astrocytes and neurons, hepcidin induces not only FPN downregulation, but also TFR1 and DMT1 downregulation [58, 59]. This is interesting since this would mean that hepcidin not only controls iron export, but iron import as well. This action of hepcidin might include an interaction with a new hepcidin receptor in astrocytes, which activates AMP-activated kinase (AMPK) intracellularly [58]. According to You et al. [54] microglia do not increase hepcidin expression in response to inflammatory stimuli, but they do increase IL-6 significantly, more so than astrocytes. Hepcidin released from astrocytes downregulates FPN expression in neurons [54, 56] (Fig. 2). There seems to be some contradiction if hepcidin actions on FPN protect neurons from iron-overload, or they cause apoptosis through increased oxidative stress [54, 56, 59–61]. Studies which suggested that hepcidin induces apoptosis in neurons through increased iron-load, were realized in models of brain ischemia and inflammation. Ischemia induces increased expression of TFR1, which increases iron import into cells [52]. Inflammation also increases iron sequestration in neurons, because it causes upregulation of DMT1 [56], which is an iron importer, though other studies contest these changes [54]. Studies that reported no change in expression of TFR1 and DMT1 during inflammation only evaluated the role of LPS during inflammation [54], but not of other important inflammatory cytokines, such as tumor necrosis factor-alpha (TNF-α). This is important since DMT1 overexpression in the brain in response to TNF-α has been consistently observed in models of inflammation in the brain [27, 56, 62]. In terms of iron transport, inflammation in the brain does seem to tip the balance in favor of increased cellular iron sequestration. An important issue stemming from Urrutia et al. study [56], is the level of FPN downregulation observed in neurons due to inflammatory stimuli; TNF-α, LPS and especially IL-6 induce a robust downregulation of FPN protein levels in neurons compared to untreated control, irrespective of hepcidin action. Similar results were obtained by Zhang et al. study where proinflammatory cytokines reduced FPN expression in dopaminegic neurons [63]. You et al. study [54] did not find any significant changes in TFR1 and DMT1 levels in neurons due to LPS treatment and attributed iron accumulation in neurons due to FPN downregulation, which could be related with marked FPN downregulation caused by inflammatory stimuli. On the other hand, Urrutia et al. did observe increase in DMT1 expression in neurons, but this was not as robust as FPN downregulation [56]. This study also found that LPS induced a less pronounced DMT1 upregulation compared to IL-6 and TNF-α. It has to be mentioned that You et al. did not examine effects of IL-6 and TNF-α on DMT1 levels. Another difference between You et al. and Urrutia et al. studies is that You et al. did not observe hepcidin overexpression in microglia and astrocytes due to LPS stimulation, but Urrutia et al. did. Also, Urrutia et al. observed increased hepcidin expression in astrocytes and microglia due to IL-6, but You et al. observed increased hepcidin expression only in astrocytes due to IL-6. These discrepancies might have occurred due to different strains of rodents or different methods of LPS treatment used by the authors. But, Urrutia et al. is in-line with You et al. study when concluding that astrocytes are the major cellular inducers of hepcidin due to inflammatory stimuli. These results suggest that at least during inflammation neurons are not the source of hepcidin expression. Another issue that remains to be resolved is if neurons increase hepcidin expression due to iron-load. Although iron-load can induce hepcidin expression in astrocytes and microglia, there is disagreement if this action occurs in neurons [56, 64]. Subtle differences observed between studies might explain these results; for example, increase in neuronal hepcidin expression was observed after 4 h of treatment with higher doses of iron therapy, whereas Urrutia et al. did not obtain these results probably due to lower levels of iron supplementation used or due to early measurements of hepcidin expression (2 h). Future research should clarify this important issue.

New research suggests that pretreatment with hepcidin downregulates markers of inflammation and oxidative stress in activated astrocytes and microglia by amyloid-β (Aβ), which protects neurons from cellular damage [65]. This is due to hepcidin ability to downregulate inflammatory stimuli. This is not the first time that hepcidin is associated with antiinflammatory response. De Domenico et al. study [66] showed that hepcidin pretreatment reduces the ability of LPS to induce cytokine production. This action of hepcidin is mediated through inhibition of IL-6 and TNF-α via suppressor of cytokine signaling 3 (SOCS3). The role of hepcidin in controlling antiinflammatory response is further strengthened by Burté et al. study [67], where higher levels of hepcidin were associated with better outcome during malaria infection. Role of hepcidin as an antiinflammatory peptide might serve as the “closing act” of the feedback loop that controls the inflammatory response. This new role of hepcidin is intriguing and it is surprising that it has not been examined more extensively.

In physiological conditions hepcidin induces downregulation of iron importers in neurons [59]. This would be the reason why hepcidin protects neurons from iron-overload [60, 61]. Neurons compared to glial cells express much higher levels of DMT1 in physiological conditions [29]. This means that in neurons cellular iron-load can be significantly affected by iron import(ers). There are suggestions that the protective effects of increased hepcidin production might occur not just as a result of hepcidin actions on neuronal cells, but also due to its actions on endothelial cells of the BBB [59]. These actions would result in decreased iron entry in brain cells. Indeed, iron-loaded astrocytes act in neighbouring endothelial cells of the BBB by reducing the expression of TFR, thus impairing iron-transport through BBB [68]. This action of astrocytes is realized through hepcidin according to in vitro animal models [68].

It is still not known how iron-load induces hepcidin expression in brain cells. It seems that membrane HJV has a redundant role in this context, since its expression is nonexistent in the brain [69, 70]. Other regulatory factors of hepcidin expression, like neogenin, are expressed in the brain, but their role in controlling hepcidin expression in brain cells is unknown [69]. The main chemical signal that induces hepcidin expression in the liver, BMP6, is expressed in the brain [71, 72]. In models with Alzheimers disease (AD), BMP6 is upregulated (compared to low levels of hepcidin observed in AD) and related with impaired neurogenesis [35, 72]. In models of ischemia BMP6 has shown to have protective effects on brain tissue compared to hepcidin [71]. This shows that BMP6 does not control hepcidin expression in models of ischemia/inflammation, but if BMP6 controls hepcidin expression in the brain in response to iron-load is still an unexplained issue. Finally, TFR2 expression has also been observed in brain tissue, but it does not seem to impact hepcidin expression [73].

## Hepcidin in brain diseases

### Hepcidin in AD and Parkinsons disease (PD)

Most of brain iron in early age is sequestered in oligodendrocytes, but with increasing age iron is more prominently deposited in astrocytes and neurons, whose iron levels are low in early age [74]. Iron is important for brain cells because it is used to maintain their metabolic needs, but also for myelin synthesis and neurotransmitter production [75].

In a study with brain samples from humans with AD taken during autopsy, levels of hepcidin and FPN were low, and the same result was observed in the accompanied rat models of AD [35]. Hepcidin levels, according to mice models, exhibited changes in later stages of the disease, but not in the early stages of the disease (Table 2). These changes seem to be secondary and related to neuronal damage. It is interesting to notice that AD is mostly associated with advanced age [75], while increased hepcidin expression has been observed in aged rats and it is still not known why [76]. On the other hand, hepcidin localization in the brain of AD patients is related closely with the localization of amyloid plaques [35].

**Table 2:** Role of hepcidin in brain diseases

Methods	Brain disease	Main results	References
Expression measured with RT-PCR, while protein levels were measured with western blotting and immunohistochemistry (rat and mice brain samples)	Focal brain ischemia/reperfusion model	Hepcidin↑ in brain tissue Knock-down of hepcidin with siRNA causes FPN↑ and ferritin↓	Ding et al. [52]
Levels of protein measured with western blotting and ELISA (mice brain samples)	Intracerebral hemorrhage (ICH) model	Brain and serum hepcidin↑ Hepc^−/−^ mice are protected from brain damage during ICH Injection of hepcidin increases brain damage Increased hepcidin inhibits BMVEC iron efflux The source of brain hepcidin are astrocytes Brain hepcidin upregulation is induced due to activation of TLR4 pathway Cellular iron-load is reduced by antagonizing local hepcidin	Xiong et al. [18]
Protein levels measured with western blotting and immunohistochemistry (rat brain samples)	Subarachnoid hemorrhage model	Hepcidin↑ Hepcidin injections increase brain damage Hepcidin knock-down with siRNA decreases apoptosis and brain damage	Tan et al. [119]
Serum protein levels measured with ELISA in 86 patients	ICH	Hepcidin↑ Higher hepcidin is associated with poorer outcome Hepcidin correlates with inflammatory markers Serum hepcidin is independently associated with mRS score^a^	Xiong et al. [114]
Serum protein levels measured with ELISA in 60 children	Acute ischemic stroke (AIS)	Hepcidin↑ Hepcidin is correlated with inflammatory markers	Azab et al. [111]
Serum protein levels measured with ELISA in 74 patients	Ischemic stroke	Hepcidin↑ Hepcidin is correlated with inflammatory markers	Petrova et al. [113]
Serum protein levels measured with ELISA in 31 patients	AIS	Hepcidin↑	Slomka et al. [112]
Protein levels measured with western blotting and immunohistochemistry in brain samples of humans and transgenic mice	Alzheimers disease (AD)	Hepcidin↓ in human samples Hepcidin↓ in mice samples in advanced stages of the disease Hepcidin staining is associated with hem-rich deposits Hepcidin distribution is observed in surviving neurons around amyloid plaques	Raha et al. [35]
Serum protein levels measured with ELISA in 52 patients	AD	Hepcidin↑ Serum hepcidin correlates with CDR-SOB^b^	Sternberg et al. [89]
Expression measured with RT-PCR and protein levels measured with western blotting and immunohistochemistry (neuronal cell cultures)	Parkinson disease model induced by 6-OHDA	Hepcidin mRNA↑ EGCG attenuates brain damage by reducing hepcidin expression	Chen et al. [94]
Expression measured with RT-PCR (neuronal cell cultures)	Parkinson disease model induced by 6-OHDA	Hepcidin knock-down offers protection from 6-OHDA neurotoxicity	Xu et al. [95]
Expression measured with RT-PCR (human brain samples)	Brain tumors	Hepcidin mRNA↓ in most tumor samples	Hänninen et al. [69]
Serum protein levels measured with ELISA method in rats	Amyotrophic lateral sclerosis	Hepcidin↑	Halon et al. [134]
Protein levels measured with immunoblotting, ELISA and immunohistochemistry in 39 patients	Restless leg syndrome (RLS)	Prohepcidin↑ in brain tissue Prohepcidin↓ in cerebrospinal fluid in early-onset disease	Clardy et al. [140]
Expression measured with RT-PCR in rat brains	Chronic mild stress	Hepcidin mRNA↑ Dalteparin lowers hepcidin expression Hepcidin expression is associated with cellular iron-overload and IL-6 expression	Farajdokht et al. [156]

*6-OHDA* 6-hydroxydopamine, *BMVEC* brain microvascular endothelial cells, *EGCG* epigallocatechin gallate, *ELISA* enzyme-linked immunosorbent assay, *FPN* ferroportin, *IL- 6* interleukin 6, *RT-PCR* reverse transcription polymerase chain reaction, *siRNA* small interfering RNA, *TLR4* toll-like receptor 4

^a^mRS score: modified Rankin Scale measures the degree of disability in patients who have suffered a stroke

^b^CDR-SOB: Clinical Dementia Rating Scale Sum of Boxes is used to quantify the severity of dementia

Increased hepcidin without the change in FPN levels shows that with advancing age hepcidin cannot change the levels of FPN expression significantly [75]. It might be that the “culprit” behind these observations is iron-load, which has been shown to induce FPN transcription. Unfortunately, AD models with hepcidin knockouts have not been studied, in order to understand the role of hepcidin in this disease [77]. These models should focus on examining the role of hepcidin in early stages of the disease and identifying the cellular source of the hepcidin, which presumably should include astrocytes or microglia.

Oxidative damage is thought to be an important pathogenic factor in AD, especially in early stages of the disease [78, 79]. It is not known which is the source of oxidative damage in early AD, but iron deposition is a strong candidate. Excess iron has been observed in AD. Hipoccampal, basal ganglia, cortical samples from AD patients show increased levels of ferritin iron [80, 81]. Amyloid plaques in AD are associated with iron deposition, as well as with iron-overloaded microglia [82]. Furthermore, iron-loading has been shown to accelerate plaque toxicity, while iron-chelation reverses these changes [83]. This is important since amyloid accumulation has been observed in a large proportion of persons without cognitive abnormalities, which means that additional factors (such as iron-induced toxicity) might turn benign amyloid aggregates into brain damaging factors. Cellular iron sequestration in the brain of AD patients could happen due to disturbance in iron transport, disruption of BBB or due to micro-hemorrhages observed in AD [84]. The β-amyloid precursor, amyloid precursor protein (APP), interacts with FPN and in this way controls iron export [85]. Loss of APP causes iron retention [85]. In AD, APP mRNA is downregulated, while it has been shown that APP stabilizes FPN in neuronal membrane [85, 86].

APP downregulation in AD is accompanied with DMT1 upregulation [87]. DMT1 is co-localized with amyloid plaques, while overexpression of APP is related with DMT1 overexpression [87]. Crucially, downregulation of DMT1 with siRNA causes downregulation of APP and reduction in secretion of Aβ [87]. Recent data suggest a potential role of hepcidin in AD pathophysiology. In-line with this suggestion is the observation from genetic studies where specific genetic variations that cause a decreased expression of FPN are a risk factor for AD [88]. On the other hand, the genetic variant that offered most protection was related with increased expression of FPN. Furthermore, in vitro studies suggest that oxidative damage, which is induced during AD, can be ameliorated with hepcidin injections [59, 60]. Hepcidin injections reduce inflammatory markers in astrocytes and microglia treated with Aβ [65]. By ameliorating two of the most important pathogenic factors in AD, like oxidative damage and inflammatory process, hepcidin could prove to be a novel therapeutic agent in AD. Also, knockdown of hepcidin in activated astrocytes by inflammatory stimuli prevents iron-load and reduces apoptosis in neurons [54]. It seems that the relationship between iron importers/exporters is important in controlling iron-load in neurons. In noninflammatory conditions, hepcidin reduces iron-load because it reduces iron-import into neurons and from blood vessels into brain parenchyma. During inflammation the increase in iron-import coupled with reduction in iron-export increases the iron-load in neurons. This means that iron-load will be ameliorated by restoring iron-export (through hepcidin knockdown). Since high levels of inflammation are related to neuronal iron-overload, it would be interesting to examine how does generally low levels of brain inflammation in AD “compete” with dose-dependent actions of hepcidin in neurons.

Hepcidin disturbance in AD has recently been suggested to have a systemic nature as well. Serum levels of hepcidin are higher in AD and are related with severity of the disease [89]. Since serum hepcidin can pass BBB, it might affect iron-export into brain parenchyma through regulation of FPN. This hypothesis needs confirmation from proper experimental AD models with systemic hepcidin knockout.

Iron dysregulation has been observed in PD patients as well. Iron-load in these patients (similar to AD patients) is localized in specific structures such as substantia nigra, red nuclei, globus pallidus, with early changes observed in substantia nigra, but not in other structures [90, 91]. In AD patients with associated PD (35–40% of patients) iron-load in substantia nigra is higher than in AD alone [92].

Inflammation and oxidative stress are both present in PD [93]. In rat models of PD induced by 6-hidroxydopamine (6-OHDA), hepcidin expression induces iron-load in neurons, while hepcidin knockdown reduces the iron-load [94, 95]. It is interestingly to notice that in rat models, 6-OHDA induces upregulation of DMT1 and hepcidin, which results in increased iron entry into cells and decreased iron export through FPN downregulation [94]. DMT1 upregulation has been observed in other animal models of PD, and crucially, in human postmortem brain samples from PD patients [96]. In addition, NEDD4 family-interacting protein 1 (Ndfip1) which is a known regulator of DMT1, is overexpressed in astrocytes of PD patients [97]. Furthermore, Ndfip1 is selectively increased in substantia nigra compared to other regions [97]. Finally, Ndfip1 expression is observed more significantly in neurons with α-synuclein deposits [97]. It seems that Ndfip1 upregulation in PD is a homeostatic mechanism which serves to protect neurons from iron toxicity. Ndfip1 upregulation by iron causes DMT1 downregulation, which protects neurons from cellular iron-overload [98]. Knockout of Ndfip1 is accompanied with cellular iron accumulation, oxidative stress and death of dopaminergic neurons [97, 98]. 6-OHDA induces DMT1 upregulation by modulating IRP proteins in neurons [99]. Further evidence concerning the role of DMT1 in PD comes from genetic studies. DMT1 mutations that impair iron-import offer neuroprotection against toxins that cause PD in animal models [96], while certain DMT1 polymorphisms increase the risk for PD [100]. The use of antioxidants reduces the oxidative damage induced by DMT1. Polyphenoles like epigallocatechin-3-gallate (EGCG) protect against 6-OHDA neurotoxicity by downregulating hepcidin and DMT1 expression and by upregulating FPN [94]. Iron accumulation in PD is associated with abnormal intracellular protein aggregates named Lewy bodies in substantia nigra [101], while similarly to AD, FPN was shown to be downregulated in PD as well [102, 103]. Overexpression of FPN in cultured dopaminergic neurons reduces iron-induced oxidative stress [103]. It is interesting to notice that while 6-OHDA induces iron accumulation in neurons, in astrocytes it induces increased iron transport in and out of the cells, which might protect neurons from increased iron-load [104].

### Hepcidin in brain ischemia

Hepcidin disturbance has been observed in brain ischemia [52]. The rationale to study hepcidin is the significant relationship that exists between iron-load and brain damage in this condition [105–108]. There is evidence that excess iron does not originate from blood, at least during focal ischemia [107].

Iron-load during ischemia worsens brain damage by increasing edema and hemorrhage, while the use of iron chelators reverses brain damage [105, 107, 108]. High heme iron intake is also related with stroke risk, while HH H63D homozygosity is a predictor of increased risk of stroke [109, 110]. Interestingly, the same mutations are a risk factor for neurodegenerative diseases [84]. Significant hepatic hepcidin disturbance can cause brain iron-overload, as it has been observed in cirrhotic patients [39]. But, in acute conditions, such as acute ischemic stroke and intracerebral hemorrhage, serum hepcidin levels are increased and they contribute to iron-load in ischemic brain [111–114]. What is more, brain damage worsens during ischemia when accompanied with increased systemic hepcidin levels, due to increased brain oxidative injury [18]. Brain hepcidin expression is also elevated during ischemia, and the source of this hepcidin are astrocytes [18]. Upregulation of hepcidin is associated with downregulation of FPN in neurons, but also with decreased iron-efflux from brain endothelial cells into plasma [18]. Studies have revealed the mechanistic pathways that induce hepcidin expression during brain ischemia; they show an important role for TLR4 and their downstream targets (IL6/STAT3 pathway) [18]. It is interesting to notice that TLR4 signalling has been consistently linked with brain damage during intracerebral hemorrhage [18, 115, 116]. But, similar to neurodegenerative diseases, iron-efflux blockade together with the increase in iron import will cause iron-overload in ischemic brain. TFR1 is upregulated in brain ischemia due to hypoxia-inducible factor 1-alpha (HIF1-α) actions [52]. Also, DMT1 upregulation is a consequence of ischemia, while its downregulation offers neuroprotection in models with brain ischemia [117]. Tanshinone IIA is a Chinese herb used in the treatment of cerebrovascular diseases. Its neuroprotective mode of action includes the reduction in the expression of TFR1 and DMT1, while at the same time it induces overexpression of FPN [118]. That is why in animal models hepcidin treatment during brain ischemia worsens iron-load and brain damage, while hepcidin knockdown ameliorates this damage [18]. Similar results were obtained in mice models with subarachnoid hemorrhage where addition of hepcidin worsened cell apoptosis, while knockdown of hepcidin with siRNA reduced apoptosis significantly [119].

### Hepcidin in multiple sclerosis (MS)

MS is a “classical” neuroinflammatory disease, characterized mainly by a dysregulated activation of adaptive immune system [120]. Iron accumulation has been evidenced early in MS and it progresses during the development of the disease [120, 121]. Iron deposition is observed in active MS lesions and blood vessels, but not in inactive lesions [120]. The source of iron in MS is supposed to be multifactorial; it can originate from degenerated oligodendrocytes, damaged blood vessels or it might occur due to dysregulation of iron transport [120]. Studies suggest that iron accumulation in MS precedes the development of atrophy [122]. Furthermore, brain iron deposition measured with MRI is a better predictor of disability than whole brain atropy [122]. In conclusion, a significant number of studies reveal an important role for iron deposition in the pathophysiology of MS, but still it is not clear if this role is of primary nature.

Iron-chelation has not proved to be a conclusive beneficial therapy in MS, suggesting local changes are more important in the pathophysiology of MS [120, 123]. In favor of this argument is the observation that serum levels of hepcidin, iron and ferritin do not change in MS [124]. Iron-chelation does not seem to be the best option to correct iron dysmetabolism in MS, since iron is needed for myelinization. A more viable option seems to be the downstream effects of iron dysregulation, like increased oxidative stress [120, 123]. This means that tackling iron dysmetabolism in MS has to be made in a local manner and with specific molecular targeting (local hepcidin?) that would not cause global changes in brain iron metabolism. Small studies have shown specific changes in TFR levels in periplaque white matter of MS patients, while experimental models have shown increased levels of DMT1 in astrocytes around MS lesions [125, 126]. Unfortunately, there are no comprehensive studies that have examined neuronal expression of TFR1 and DMT1 in relation to FPN in MS.

Similarly to AD and PD, inflammatory and hypoxic conditions are present in MS and thought to contribute to iron dysmetabolism [120]. But, if local hepcidin has a role in MS is not known, especially with the lack of studies that have examined the role of this peptide in MS.

### Hepcidin in amyotrophic lateral sclerosis (ALS)

ALS is a motor neuron disease associated with changes in iron metabolism [127]. Ferritin and serum iron are increased in patients with ALS [128, 129]. Furthermore, ferritin levels are correlated with disease duration [128]. Iron deposition in motor cortex of ALS patients is not primary related to disease pathogenic mechanisms. Microglia in ALS are reported to become iron-loaded due to scavenging of debris created during the primary process of neuron damage [130, 131]. This increase in iron deposition is partly related to increased TFR1 expression in glial cells [130]. Similarly, neuron iron-load in ALS is related with increase of another iron importer, that is, DMT1 isoform, which expression is not under IRE control, but is under control of inflammatory signals [130]. This is important because inflammation has been observed as an important pathogenic factor in ALS [132]. On the other hand, the expression of FPN in ALS though increased, is still lower than that of DMT1, which would favor iron deposition in cells [130]. Iron-overload seems to be an important player in neuronal damage, since iron-chelation increases life-span and offers neuroprotection in transgenic mice [130, 133]. Though central nervous system (CNS) actions of hepcidin in ALS have not been evaluated, in transgenic models of this disease serum hepcidin increase is accompanied by FPN downregulation in muscles [134]. But, increased systemic hepcidin cannot explain why FPN downregulation is observed only in certain muscles in ALS. Finally, studies need to address the role of systemic and brain hepcidin in iron dysregulation in ALS through its effects on iron sequestration in brain cells.

### Hepcidin in brain cancer

Hepcidin levels in different brain cancers are generally lower compared to normal tissue with a fairly high degree of heterogeneity found between tumors [69]. But, global changes in the brain might not reveal the exact picture in brain tumors compared to specific cell actions. Glioblastoma stem-like cells (GSCs) are at the apex of the hierarchical organization of cells in glioblastoma [135]. These cells can initate, promote and renew brain tumor proliferation. Their high activity in brain cancer has to be maintained with rich supply of nutrients such as iron. This is in-line with results that have shown higher iron-uptake from brain tumor tissue compared to normal tissue [19]. GSCs have the ability to preferentially “steal” iron from their environment by increasing TFR1 expression, that is, iron import [136]. This is probably the reason why hypoxia reverses antiproliferative effects of iron-chelation in brain cancer, since hypoxia in the brain does induce TFR1 expression [52, 137]. But, GSCs might also use hepcidin to increase their cellular iron depots. GSCs show increased levels of ferritin, which is related with increased STAT3 phosphorylation [136]. STAT3 is a known intracellular signaling molecule that induces hepcidin expression [17]. Whether GSCs use hepcidin to increase iron sequestration is an intriguing idea, but has to be validated by future research.

### Hepcidin in restless leg syndrome (RLS)

RLS is a neurological condition with a highly heritable component. It is characterized with an urge to move lower limbs, while many patients show signs of iron deficiency [138]. Iron depots in the brain of RLS patients are low and related with higher expression of brain hepcidin [139, 140]. Low levels of iron in the brain of RLS patients persists even in the presence of HH [141]. Furthermore, transferrin receptor expression in the brain microvasculature of RLS patients is low, suggesting low iron transport across the BBB [142]. Still, the role of hepcidin is unclear especially since high levels of brain hepcidin in RLS are accompanied with low levels of hepcidin in CSF [140]. It is interesting to notice that in vitro results show that hepcidin reduces iron-overload only in iron-loaded neurons [60, 61]. It might be that higher hepcidin levels in the brain of RLS patients occur as a reactive response to protect neurons from iron deficiency by reducing the activity of FPN, though this idea needs confirmation.

### Potential role of hepcidin in other brain conditions

Huntington disease (HD) is a genetic disorder characterized with mutation of the gene encoding for Huntington protein (Htt), which results in the creation of a mutant Htt with toxic properties [143]. HD studies show early destruction of neurons and astrogliosis in the striatum with progressive involvement of cortex and hipoccampus as the disease develops [143]. There is evidence of increased brain iron deposition in this disease, even in the early phases of the disease [144–146], while expression of Htt in animal models has been shown to be associated with cellular iron homeostasis [143]. There is no compelling evidence that iron-overload in specific structures can serve as the initiator of the disease, but the increase of iron depots as the disease develops shows a role for iron in later stages of the disease. If this role is secondary due to neuronal damage, or it contributes directly in disease progression is still unknown. Although there are no studies that have examined the role of hepcidin in HD, the efficacy of iron-chelation therapy and the observed reactive upregulation of FPN due to neuronal iron-overload suggests that hepcidin downregulation might attenuate neuronal damage [147].

Iron dysregulation has been observed consistently in different organs in Friedreich ataxia (FA) [148]. This disease affects CNS by causing neurodegenerative damages in dentate nuclei of the cerebellum, dorsal root ganglia, but also in cerebrum, thalamus and other structures [149]. FA is caused by a defective frataxin, which main functions include involvement in iron-sulfur cluster formation and in iron delivery to ferrochelatase [148]. Although some observations did find iron accumulation in dentate nuclei in FA patients, other studies have revealed a pattern of iron redistribution, rather than iron accumulation in FA [150]. Studies suggest that iron dysregulation in FA is not needed for neurodegeneration to occur, while animal models reveal tissue-specific damages due to frataxin deficiency [148, 151]. These differences are related with levels of frataxin expression, where most of the damage is observed in tissues with higher expression of frataxin, such as the heart and dorsal root ganglia [151]. In the heart, inflammatory infiltrate produces hepcidin and has been proposed as one of the pathogenic mechanisms of heart damage in FA [152]. Frataxin deficiency in animal models can cause a strong inflammatory reaction in Schwann cells, which are known to enwrap neurons of dorsal root ganglia [153]. These neurons are frequently affected in FA and are characterized with iron dysregulation and inappropriate myelination [154]. But, hepcidin expression in these cells has not been studied in models of FA, therefore it is not known what role, if any, does hepcidin have in the pathophysiology of neurodegeneration in FA.

## Conclusion

Although data about the role of systemic and local hepcidin in the brain are scarce compared to liver, recent research suggests that hepcidin has important functions in proper brain functioning. Hepcidin in the brain is expressed in low levels, with protein levels being more prominently higher than mRNA expression, suggesting that much of hepcidin in the brain originates from the liver. Studies suggest a high probability that plasma hepcidin crosses BBB, but the real mechanisms behind this transport are not known. Although we know the cellular source of hepcidin in the brain, its regulation is still not well understood. Studies suggest that astrocytes and microglia increase hepcidin expression in response to inflammatory stimuli, and probably to iron-load. Data concerning hepcidin expression in neurons in basal conditions are contradictory and still not well understood, though hepcidin expression has been consistently observed in neurons exposed to inflammatory stimuli. Nevertheless, hepcidin does not seem to be important for iron homeostasis in basal conditions, since hepcidin knockout models do not show significant brain iron-overload. Studies suggest that hepcidin therapy protects neurons from iron-overload, but when hepcidin overexpression is observed in the presence of significant inflammatory activity then hepcidin has deleterious effects in neuronal function by inducing iron-overload in neurons. It seems that inflammatory signaling in the brain tips the balance in favor of increased iron-import and cellular iron sequestration. Inflammatory actions accompanied with hepcidin effect in blocking iron-export, further worsens the iron-overload observed in neurons. When inflammation is not present, hepcidin protects from iron-overload due to its role in reducing iron-entry into neurons. In experimental mice models this has already been done with the use of recombinant viruses that contain hepcidin encoding DNA. Recent data suggest that pretreatment with hepcidin has protective functions in neurons. This observation is based on the ability of hepcidin to reduce inflammatory signaling in pretreated cells. This exciting data should be further elucidated.

During ischemia and inflammatory conditions hepcidin expression (mostly from astrocytes) is related with brain damage. The most important data come from models with brain hemorrhage, where hepcidin overload contributes to brain damage. Blocking local and systemic hepcidin in these conditions ameliorates brain damage. This means that hepcidin therapy can be used to ameliorate brain damage during ischemia. Hepcidin anatagonists are available, and although they have been used mainly for inflammatory anemia characterized with high levels of hepcidin [155], their use in brain tissue has shown that it can downregulate hepcidin expression [156].

There is increasing evidence, albeit circumstantial, that hepcidin might play a role in the pathogenic mechanisms in neurological conditions like AD and PD. Direct role of hepcidin in these conditions, especially in early stages of the disease, has not been studied. Hepcidin disturbance has been observed in other conditions as well, like in brain cancer, where levels of local hepcidin are generally low, but the importance of this observation remains to be unveiled.

In conclusion, hepcidin is expressed in the brain and has important homeostatic functions. Manipulating hepcidin could prove to be a novel therapeutic option in tackling brain damage, especially during brain ischemia/inflammation and iron-overload.

## Abbreviations

6-OHDA: 6-hidroxydopamine
AD: Alzheimers disease
AMPK: AMP-activated kinase
APP: amyloid precursor protein
Aβ: amyloid-β
ALS: amyotrophic lateral sclerosis
BBB: blood–brain barrier
BMPR: BMP receptor
BMP6: bone morphogenetic protein 6
BMVECs: brain microvascular endothelial cells
CNS: central nervous system
CSF: cerebrospinal fluid
CP: ceruloplasmin
DMT1: divalent metal transporter 1
EGCG: epigallocatechin-3-gallate
ERFE: erythroferrone
FPN: ferroportin
FA: Friedreich ataxia
GSCs: glioblastoma stem-like cells
HH: hemochromatosis
HJV: hemojuvelin
*HAMP*: hepcidin antimicrobial peptide
Heph: hephaestin
HD: Huntington disease
Htt: Huntington protein
HIF1-α: hypoxia-inducible factor 1-alpha
IL-6: interleukin-6
IRE/IRP: iron regulatory element/iron regulatory protein
JAK2/STAT3: janus kinase 2/signal transducer and activator of transcription 3
LPS: lipopolysaccharide
LSECs: liver sinusoidal endothelial cells
MT2: matriptase 2
MS: multiple sclerosis
Ndfip1: NEDD4 family-interacting protein 1
NTBI: non-transferrin bound iron
PD: Parkinsons disease
PCBPs: poly-(rC)-binding proteins
RLS: restless leg syndrome
RPE: retinal pigment epithelium
SMAD: s-mothers against decapentaplegic
SOCS3: suppressor of cytokine signaling 3
TLR4: toll-like receptor 4
Tf: transferrin
TFR1: transferrin receptor 1
TNF-α: tumor necrosis factor-alpha
